# Predictors of successful trial of labor after cesarean section (TOLAC) in women with one prior transverse cesarean section at Tertiary Hospitals in northwest Ethiopia: a multicenter study

**DOI:** 10.1186/s12884-024-06432-z

**Published:** 2024-04-05

**Authors:** Gizachew Aynalem Tegegne, Bayew Kelkay Rade, Ayenew Engida Yismaw, Worku Taye, Berihun Agegn Mengistie

**Affiliations:** 1 Department of Midwifery, Debremarkos Referral Hospital, Debremarkos, Ethiopia; 2 Department of General Midwifery, School of Midwifery, College of Medicine and Health Sciences, University of Gondar, Gondar, Ethiopia; 3 Department of Clinical Midwifery, School of Midwifery, College of Medicine and Health Sciences, University of Gondar, Gondar, Ethiopia

**Keywords:** Ethiopia, Prior cesarean section, Tertiary hospitals, Trial of labor after cesarean section, TOLAC

## Abstract

**Background:**

Trials of labor after cesarean section is the preferred strategy to decrease the cesarean delivery rate and reducing complications associated with multiple cesarean sections. The success rate of trials of labor after cesarean section and associated factors have not been well documented in Ethiopia. Hence, this study was aimed to determine the success rate and factors associated with the trial of labor after one cesarean section in five Comprehensive Specialized Hospitals located in northwest Ethiopia.

**Methods:**

An institutional-based cross-sectional study was conducted among 437 women who came for the trial of labor from December 1, 2021, to March 30, 2022. All women who fulfilled the eligibility criteria were included to this study. Data was collected using structured and pre-tested questionnaire. Then, the data was entered into Epi Data 4.6 software and exported to SPSS version 26 for analysis. To identify the variables influencing the outcome variable, bivariable and multivariable logistic regression analyses were conducted. The model’s fitness was checked using the Hosmer-Lemeshow goodness of fit test, and an adjusted odds ratio with a 95% confidence interval was used to declare the predictors that are significantly associated with TOLAC.

**Results:**

The success rate of the trial of labor after one cesarean section was 56.3% (95% CI, 51.3%, 61.2%). Maternal age ≥ 35 years (AOR: 3.3, 95% CI 1.2, 9.3), the fetal station at admission ≤ zero (AOR: 5. 6, 95% CI 3.3, 9.5), vaginal delivery before cesarean section (AOR: 1.9, 95% CI 1.2, 3.2), and successful vaginal birth after cesarean delivery (AOR 2.2, 95% CI 1.2, 4.1) were found to have a significant association with the success rate of trial of labor after cesarean section.

**Conclusions:**

In this study, the success rate of the trial of labor after a cesarean section was low as compared to the ACOG guideline and other studies in different countries. Therefore, the clinicians ought to offer counsel during antenatal and intrapartum period, encourage the women to make informed decision on the mode of delivery, and the practitioners need to follow fetal and maternal conditions strictly to minimize adverse birth outcomes.

## Introduction

Trial of labor after cesarean (TOLAC) is a planned attempt to allow labor in women who have had one previous cesarean birth, regardless of the outcome of the previous cesarean [1, 2]. This approach allows women who want a vaginal delivery to achieve that goal—a vaginal birth after cesarean delivery [2].

Globally, the trial of labor after cesarean delivery is considered a reasonable, safe option that is highly effective at reducing the overall CS rate and obstetric complications [3]. The overall success rate of TOLAC among ACOG’s members was 60-80%, resulting when the first cesareans were performed for non-repeating indications [2]. The successful rate of TOLAC was reported 76.6% in Canada [4], 80.7% in Taiwan [5], 62.8% in Norway [6], 61.8% in Nigeria [7], 57.6% in the DRC [8] and 69.4% in Addis Ababa, Ethiopia [9]. In Sub-Saharan Africa, a meta-analysis and systematic review revealed a 75% success rate for TOLAC [10]. To lower the rising rate of cesarean sections, different organizations and expert panels have advocated for women who meet certain criteria to attempt TOLAC [2, 11].

For TOLAC, the following requirements must be met: one prior cesarean section; a lower transverse uterine incision during the prior CS; a cephalic presentation; and the absence of any other uterine scars, such as myomectomy [2, 12]. In addition, the facility where a woman with a previous scar can undertake TOLAC should have the resources to conduct an emergency repeat cesarean section (ERCS) within a reasonable time frame, ideally, within ten minutes of the decision. These resources include a skilled clinician who can monitor labor and perform an ERCS, a clinician who can administer obstetric anesthesia, nursing personnel to assist with the ERCS, and a clinician who can perform neonatal resuscitation if necessary [2, 12].

A successful TOLAC is associated with reduced the risk of multiple CS, hysterectomy, bladder injury, anesthesia-related complications, reducing abnormal placenta implantation, decreasing blood loss, postpartum infections, intra-abdominal adhesion, and deep vein thrombosis, pulmonary embolisms, surgical site infections, helping clear the baby’s lungs, and shorter hospital stay [13–15]. It lowers the country’s overall CS rate while also reducing maternal morbidity and mortality in the next pregnancy [16, 17]. However, failed TOLAC increases the aforementioned maternal morbidity, uterine rupture (less than 1%), and unfavorable perinatal outcomes [18, 19]. These risks can be minimized by careful monitoring and adhering to institutional guidelines [20].

Predicting the likelihood of successful TOLAC has been clinically important to reduce TOLAC complications [21]. The predictive factors for a successful TOLAC include vaginal delivery before CS, spontaneous onset of labor, history of vaginal birth after CS, favorable cervix (higher Bishop score), fetal station, a non-recurrent indication of previous CS, maternal age less than 40 years, inter-delivery intervals ≥ 18 months, residence, rupture of membrane, fetal weight less than 4 kilograms, gestational age, and singleton pregnancy [21–24].

Despite TOLAC being practiced in Ethiopia, little is known regarding the success rate and contributing factors of the trial of labor after cesarean section. In Ethiopia, there is limited evidence, particularly in the study area. Therefore, in light of the benefits of the success rate of TOLAC, and scarcity of research, the purpose of this study was to determine the success rate and factors associated with TOLAC in mothers who had one lower transverse previous CS.

## Methods

### Study design, period and setting

An institutional-based cross-sectional study design was conducted at Comprehensive Specialized Hospitals located in Northwest Ethiopia from December 1, 2021 to March 30, 2022. The Amhara regional state is located in the north-western and north-eastern parts of Ethiopia. As per the Amhara regional state health office, there were eight Comprehensive Specialized Hospitals. This study was conducted among mothers who came for a trial of labor in five randomly selected comprehensive specialized hospitals found in northwest Ethiopia. These were the University of Gondar (UoGCSH), Felege-Hiwot (FHCSH), Tibebe-Ghion (TGCSH), Debre-Markos (DMCSH), and Debre-Tabor comprehensive cpecialized hospitals (DTCSH). UoGCSH is one of the country’s largest teaching hospitals, located in Gondar, Ethiopia. It’s located in Gondar City, approximately 750 km from Addis Ababa, Ethiopia’s capital city. The second hospital, FHCSH, is located in Bahir Dar, 565 kilometers northwest of Ethiopia’s capital. It has different departments, and it serves an estimated eight million people residing in urban and rural parts of North West Ethiopia per year. There were about five obstetricians and gynecologists and 63 midwives. The obstetric ward is the one that gives on average 6480 delivery services per year. TGCSH is a teaching hospital affiliated with Bahir Dar University’s College of Medicine and Health Sciences and located in Bahir Dar, Ethiopia. There were about 3,400 deliveries per year. There were 20 obstetricians, 56 residents, and 72 midwives currently working in the obstetrics and gynecology department.

Debre Markos town is the capital of the East Gojjam zone which is 300 km from Addis Ababa and 265 km from Bahir Dar, the capital city of Amhara Regional State. DMCSH is the only tertiary hospital, and providing service for more than 5 million people per year. In the maternity department, there were seven obstetrics and gynecology specialists, 46 midwives, one emergency surgeon, three clinical midwifery specialists, and 14 general practitioners. Debre Tabor town is located 666 km from Addis Ababa, the capital city of Ethiopia. DTCSH is located in Debre Tabor town and serves more than 2.4 million people in its catchment area. There were five obstetricians and gynecologists, 44 midwives, five clinical midwifery specialists, and three emergency surgeons.

### Study participants and eligibility criteria

All pregnant women who had one previous lower transverse cesarean section and who came for the trial of labor at comprehensive specialized hospitals in northwest Amhara during the study period or data collection period. Women who had one prior lower uterine segment transverse cesarean section scar and the current pregnancy with a single, live fetus with vertex presentations, who have reached 28 weeks of gestation or seven months of amenorrhea, volunteer to attempt TOLAC, and an inter-delivery interval of 18 months or above were included in the study. Whereas, a pregnant woman with an estimated fetal weight greater than 4000 grams or non-reassuring fetal heart rate patterns (NRFHRPs) in the intrapartum period was excluded from the study.

### Sample size determination and sampling technique

The sample size was determined by using the single population proportion formula by considering the 45% success rate of TOLAC taken from the previous study conducted in Attat Primary Hospital, Gurage Zone, Ethiopia [25], a 95% level of confidence, and a 5% margin of error. Thus, the calculated sample size was 380. Lastly, by considering a 15% non-response rate, the minimum adequate sample size became 437. When a survey was done to assess the numbers of pregnant mothers admitted for the trial of labor after one prior CS per month in five comprehensive specialized hospitals, it was 97 (ninety-seven). Then, all women who fulfilled the eligibility criteria were included in the study. Mothers who had one previous CS were identified and interviewed with their agreement, and data was collected starting after being admitted to the labor ward. So, 437 participants were found in in the study period and data were gathered from them, including 119 from Debre-Markos, 97 from Felege-Hiwot, 86 from the University of Gondar, 72 from Tibebe-Ghion, and 63 from Debre-Tabor Comprehensive Specialized Hospitals.

### Study variables

Success rate of TOLAC was the outcome variable, whereas socio-demographic factors (age, residence, marital status, religion, occupation, educational level), present and past obstetric history (parity, gravidity, antenatal care follow-up, spontaneous labor, indication of the previous CS, inter-delivery interval, prior vaginal deliveries, vaginal birth after cesarean section (VBAC), station of fetal station at admission, cervical dilatation at admission, rupture of membrane, artificial rupture of membrane, estimated fetal weight, and gestational age) were independent variables.

### Data collection tool and data quality assurance

A structured and pre-tested data collection questionnaire was prepared following a comprehensive review of existing literature with the same study aims [21, 22, 24–27]. The tool was initially organized in English and translated into Amharic and back into English by language experts to guarantee uniformity. Finally, the data collection was conducted using the Amharic version, which is the first language of the study participants. The questionnaire consists of socio-demographic characteristics and present and past obstetrical histories. Data were collected by ten trained bachelor’s degree midwives who had at least one year’s work experience, and five senior (more than 5 years’ experience) BSc midwives were assigned for supervision. Mothers were interviewed when a trial of labor started, and the final was recorded at the end of labor. Moreover, the questionnaire was pre-tested on 5% of the study subjects at Motta General Hospital in order to check for completeness, response, language clarity, and necessary modifications to the tool. Besides, one-day training was given to the data collectors and supervisors on the objective of the research, the content of the tool, the data collection process, ethical considerations, and the completeness of the data. Finally, the collected data were checked for completeness and given a unique code by the principal investigator before data entry.

### Data management and analysis

After data collection was checked for completeness, it was coded and entered into Epi-Data 4.6 software, and then exported to Statistical Package for Social Science (SPSS) version 26 for analysis. The results were presented in tables and graphs by cross-tabulating independent variables with the TOLAC success rate. A bi-variable logistic regression analysis was conducted to assess the association between the success rate of TOLAC and its predictors, which fulfilled the assumption of a chi-square distribution. Then, variables whose *p*-value was less than 0.25 were fitted into a multivariable logistic regression model. In the final model, variables with an adjusted odds ratio (AOR) with a *p*-value less than or equal to 0.05 at a 95% confidence interval (CI) were considered statistically significant predictors. Multi-collinearity between the independent variables was checked using the variance inflation factor (VIF), which indicates that there was no significant multi-collinearity since all variables have a VIF <10. Finally, the goodness-of-fit of the model was also examined by Hosmer and Lemeshow and was found to be good.

## Results

### Socio-demographic characteristics of respondents

During the study period, 437 women participated in a trial of labor following cesarean section, with a mean age of 29.9 (± 3.77 standard deviation) and a minimum and maximum age of 22 and 40 years, respectively. The vast majority of responders (94.1%) were Orthodox Christians, and they were all married. In terms of education, 25.6% of the participants could not read or write. More than half of the participants (57.4%) came from urban, and more than half (52.9%) were referred by health centers (Table 1).

**Table 1:** Socio-demographic factors of women who came for the trial of labor after cesarean section at comprehensive specialized hospitals in Northwest Ethiopia 2022. (*n* = 437)

Variable	Categories	Frequency	Percent
Age in years	<25	58	13.3
	25-34	310	70.9
	≥35	69	15.8
Religion	Orthodox	411	94.1
	Muslim	17	3.8
	Protestant	9	2.1
Occupation	Housewife	239	54.7
	Merchant	84	19.2
	Government employee	85	19.5
	Private employee	22	5.0
	Daily labor	7	1.6
Level of education	Unable to read & write	112	25.6
	Able to read &write	25	5.7
	Primary (1-8)	105	24.0
	Secondary	106	24.3
	College and above	89	20.4
Residence	Urban	251	57.4
	Rural	186	42.6
Source of referral	Health center	231	52.9
	Hospital	97	22.2
	Self or private	109	24.9

### Success rate of trial of labor, obstetric and fetal related characteristics

In this study, the TOLAC success rate was reported to be 246 (56.3%). In terms of mode of delivery, 51.5% of the individuals used spontaneous vaginal delivery (SVD). Nearly half of the total participants (49.7%) were gravida two; all pregnancies were planned and received ANC follow-up during the current pregnancy. The vast majority of respondents (98.4%) had no prior experience with abortion (Table 2).

**Table 2:** Successful TOLAC, obstetric and fetal-related characteristics of the current pregnancy among mothers who had attended a trial of labor after a cesarean section at comprehensive specialty hospitals in Northwest Ethiopia, 2022 (*N* = 437)

Variables	Categories	Frequency	Percent
Gravidity	Two	217	49.7
	Three	106	24.2
	≥Four	114	26.1
Abortion	Yes	7	1.6
	No	430	98.4
Parity	≤ two	324	74.1
	Three	74	16.9
	≥ Four	39	8.9
Number of antenatal care follow-ups (*N*=437)	Two	60	13.7
	Three	196	44.9
	Four	132	30.2
	≥ five	49	11.2
Place of ANC follow-up	Health center	361	41.0
	Hospital	329	37.3
	Private facility	191	21.7
Gestational age calculated by LNMP or by early ultrasound scanning result	<37 weeks	12	2.7
	37-41.6 Weeks	345	78.9
	≥ 42 weeks	21	4.8
	Unknown	59	13.6
Estimated fetal weight at admission by Johnson formula	<2500 grams	14	3.2
	2500-3999 grams	420	96.1
	≥ 4000 grams	3	0.7
Cervical dilatation at admission	<4 cm	200	45.8
	≥4 cm	237	54.2
Station at admission (At first evaluation)	<0 (below 0)	291	66.6
	≥0 (0 and above)	146	33.4
Membrane status at admission	Intact	292	66.8
	Rupture	145	33.2
Artificial rupture VS rupture of membrane done, (*N*= 292)	Yes	219	75.0
	No	73	25.0
Meconium status of amniotic fluid at the end of labor	Clear	370	84.7
	Grade 1	47	10.7
	Grade 2	11	2.5
	Grade 3	9	2.1
Duration of labor from admission to delivery	<4 hrs	175	40.0
	4-12 hrs	235	53.8
	≥13 hrs	27	6.2
Type of health professional who follows labor	Midwife	254	58.1
	Internship student	183	41.9
The educational level of midwives who followed labor (*N*= 254)	Diploma	46	18.1
	Degree	155	61.0
	MPH/MSc	53	20.9
Mode of delivery after a trial of labor	SVD	225	51.5
	Vacuum	17	3.9
	Forceps	4	0.9
	Cesarean section	191	43.7
Neonatal outcome of the current pregnancy	Alive	436	99.8
	Death	1	0.2
First-minute APGAR score	0-3	1	0.2
	4-6	34	7.8
	7-10	402	92
Fifth-minute APGAR score	0-3	1	0.2
	7-10	436	99.8
Sex of the neonate	Male	225	51.5
	Female	212	48.5

*SVD* Spontaneous vaginal delivery

### Indications of a cesarean section after trial of labor for the current pregnancy

Of the total women who were eligible for a trial of labor after one prior cesarean section, 191 (43.7%) had failed TOLAC. The most common reason for a cesarean section was prolonged latent first stage of labor/LFSOL (13.5%), followed by non-reassurance fetal heart rate pattern or non-reassuring fetal heart rate patterns (NRFHRP) (10.5%).

### Past obstetric-related factors of study participants

Concerning prior CS indications, 199 (45.5%) of the procedures were performed to detect a non-reassurance fetal heart rate rhythm. This survey also discovered that 297 (68%) of respondents had an interpregnancy gap of 25-60 months. Before main CS, 193 (44.2%) of the study participants had a history of spontaneous vaginal birth, and 75.1% experienced one spontaneous vaginal delivery. One-fourth (25.6%) of the patients had a successful TOLAC history, while the majority (92.9%) had at least one VBAC history (Table 3).

**Table 3:** Previous obstetric factors among women who had attended the trial of labor after cesarean section at comprehensive specialized hospitals in Northwest Ethiopia, 2022 (*N* = 437)

Variables		Categories	Frequency	Success TOLAC *N*=246	Failed TOLAC *N*=191
Indication of previous cesarean section	NRFHRP	Yes	199 (45.5)	114	85
		No	238 (54.5)	132	106
	Failed induction	Yes	56 (12.8)	27	29
		No	381 (87.2)	219	162
	Mal-presentation	Yes	56 (12.8)	34	22
		No	381 (87.2)	212	169
	APH	Yes	38 (8.7)	25	13
		No	399 (91.3)	221	178
	CPD	Yes	22 (5)	13	9
		No	415 (95)	233	182
	Twin pregnancy	Yes	21 (4.8)	11	10
		No	416 (95.2)	245	171
	Cervical dilatation disorder	Yes	13 (3)	7	6
		No	424 (97)	235	189
	Grade 3 meconium	Yes	12 (2.7)	5	7
		No	425 (97.3)	241	184
	Mal-position	Yes	8 (1.8)	4	4
		No	429 (98.2)	242	187
	Unknown	Yes	12 (2.7)	6	6
		No	425 (97.3)	240	185
Inter-delivery interval	<25 months		98 (22.4)	55	43
	25-60 months		297 (68)	165	132
	≥60 months		42 (9.6)	26	16
History of Previous SVD	Yes		193(44.2)	138	55
	No		244 (55.8)	108	136
Number of previous SVD (*N*=193)	One		145 (75.1)	105	40
	Two		35 (18.1)	24	11
	≥three		13(6.8)	9	4
History of successful TOLAC	Yes		112 (25.6)	89	23
	No		325 (74.4)	157	168
Number of VBAC (*N*=112)	One		104 (92.9)	84	20
	≥ Two		8 (7.1)	5	3

*CS* Cesarean section, *SVD* Spontaneous vaginal delivery, *VBAC* Vaginal birth after cesarean section

### Factors associated with success rate TOLAC

In the bivariable logistic regression analysis, women’s age, educational status, place of residence, parity, gestational age, cervical dilatation at admission, station on admission, membrane status at admission, duration of labor during labor, history of vaginal delivery before primary cesarean section, and history of VBAC were statistically associated with the success rate of TOLAC (*p*-value < 0.25). However, in the multivariable logistic regression analysis, only maternal age 35 years old or older, station on admission, vaginal delivery before primary cesarean section, and history of VBAC were all significantly associated with the success rate of TOLAC (*p*-value ≤ 0.05).

This study shows that women whose age was 35 years old or older were three times more likely to have a successful trial of labor after a cesarean section (AOR: 3.3; 95% CI: 1.2, 9.3) than their counterparts. Regarding the progress of labor, low station of fetal head (less than or equal to zero) were five times more likely to have a success rate of TOLAC (AOR 5.6; 95% CI: 3.3, 9.5) than the station higher than zero at admission. Besides, mothers who experienced vaginal birth before the first CS (AOR: 1.9, 95% CI: 1.2, 3.2) were two times more likely to succeed in TOLAC as compared to their counterparts. Lastly, women who had previously had a successful TOLAC were 2.2 (AOR: 2.2, 95% CI: 1.2, 4.1) times more likely to give a successful trial of labor following a cesarean section (see Table 4).

**Table 4:** Bivariable and multivariable logistic regression analysis for the successful TOLAC among women who had attended trials of labor after cesarean section at comprehensive specialized hospitals in Northwest Ethiopia, 2022 (*N*= 437)

Variable with Category	TOLAC status		COR (95%CI) (Lower, upper)	AOR (95%CI) (Lower, upper)
	Success	Failure		
Age				
<25 years	24	34	1	1
25-34 years	164	146	1.6 (0.9, 2.8)	1.4 (0.7, 2.6)
≥35 years	58	11	7.47 (3.3, 17.1)	3.3 (1.2, 9.3)*
Level Education				
Unable to read & write	70	43	1	1
Able to read & write	15	10	0.9 (0.4, 2.2)	0.5 (0.2, 1.4)
Primary (1-8)	49	57	0.5 (0.3, 0.9)	0.8 (0.4, 1.4)
Secondary (9-12)	56	49	0.70 (0.4, 1.2)	1.03 (0.6, 1.9)
College & above	56	32	1.1 (0.6, 1.9)	1.6 (0.8, 3.2)
Residence				
Urban	135	116	1	1
Rural	111	75	1.3 (0.9, 1.9)	1.2 (0.6, 2.1)
Number of parities				
≤two	158	166	1	1
Three	54	20	2.9 (1.6,5.0)	0.6 (0.2, 1.5)
≥Four	34	5	7.1 (2.7,18.7)	0.8 (0.2, 3.6)
Gestational age				
<37 weeks	5	7	1	1
37-40 weeks	189	156	1.7 (0.5, 5.5)	0.9 (0.2, 3.2)
>40 weeks	15	6	3.5 (0.8, 15.5)	1.8 (0.3, 10.3)
Unknown GA	37	22	2.4(0.7, 8.3)	1.1 (0.3, 4.8)
Cervical dilatation at admission				
<4 cm	94	106	1	1
≥4 cm	152	85	2.0 (1.4, 3.0)	1.0 (0.6, 1.7)
Station at admission				
Above zero (> 0)	134	157	1	1
Zero and below zero (≤ 0)	112	34	3.9 (2.5, 6.0)	5.6 (3.3, 9.5)**
Membrane status at admission				
Intact	147	145	1	1
Rupture	99	46	2.1 (1.4, 3.2)	1.4 (0., 2.4)
Duration of labor after admission				
<4 hrs	101	74	2.0 (0.9, 4.5)	0.6 (0.2, 1.5)
4-12 hrs	134	101	1.9 (0.9, 4.3)	1.2 (0.5, 2.9)
>12 hrs	11	16	1	1
VD before CS				
Yes	138	55	3.2 (2.1, 4.7)	1.93 (1.2, 3.2)*
No	108	136	1	1
History of VBAC				
Yes	89	23	4.1 (2.5, 6.9)	2.2 (1.2, 4.1)*
No	157	168	1	1

*SVD* Spontaneous vaginal delivery, *VBAC* Vaginal birth after cesarean delivery, *AOR* Adjusted odds ratio, *COR* Crude odds ratio

Model fitness test (Hosmer-Lemeshow) =0.46

1= stands for reference, *Stands for *P* <0.05, **P <0.01

## Discussion

This study included 437 participant women who had one previous cesarean section scar and attempted vaginal birth in the current pregnancy. In the present study the success rate of TOLAC found to be 56.3% (95% CI: 51.3, 61.2). This finding was consistent with the study findings of 53.1% in Nigeria [23], 57.6% in the Democratic Republic of the Congo [28], 57% in China [29], and 60% in Thailand [30]. However, it was higher than the study’s findings conducted in 41% and 45% in Ethiopia, [24, 25], 31.6% in Egypt [31], 45.1% in Rwanda [16], 33.8% in Nigeria [22], and 25.6% in Iraq [32]. This disparity could be due to the variation in study settings and study periods. Another possible justification might be a variation in maternal pelvic capacities or estimated fetal weight.The finding of the current study was also lower than the study findings conducted in, 69.4% in Ethiopia [9], 62% in India [20], 62.8% in Norway [6], 91% in Iran [33], 74.6% in Italy [34], 88.6% in Japan [35], 72.9% in Finland [36], and 74% in United States [37]. This discrepancy might be due to the fact that variation in hospital settings or protocols for TOLAC across countries, the availability of modern equipment during labor and delivery, and socioeconomic status. The other possible reason could be related with the presence of experienced health professionals (obstetricians, midwives, anesthetists, or other health professionals) and antenatal counselling regarding the alternative mode of delivery including TOLAC. TOLAC is a reasonable strategy to minimize the morbidity associated with rising Caesarean Section. However, it’s also related with a higher risk of uterine rupture, neonatal asphyxia, and perinatal death than elective repeated CS [38].

Our study demonstrated that the major predictors for the success rate of TOLAC including maternal age 35 years old or older, station of fetal head on admission, history of vaginal delivery before primary cesarean section, and history of VBAC. Thus, women aged 35 years or older were three times more likely than their counterparts to give a vaginal birth after a cesarean section. This is in agreement with the study conducted in Ethiopia’s Harari region [39], but it contradicts the findings of the studies conducted in Ambo Town, Central Ethiopia [27], and China [29]. The likely explanation could be that when the woman’s age increases, so does her gravidity and parity; in this case, previously vaginal delivery and history of VBAC also increase. So, the pelvis was tested, the progress of labor might be facilitated, and the success rate of TOLAC increased.

Fetal stations at zero and lower the level of zero) positively influenced the success rate of TOLAC. This study was supported by the studies conducted in Addis Ababa, Ethiopia [9] and West Kazakhstan [26]. The plausible explanation is that as the station of fetal head advances, the occurrence of protracted or arrested cervical dilatation is rare. The other possible explanation could be that the lower fetal head favorable for an artificial rupture of the membrane. This facilitates uterine contraction and the progress of labor through the release of prostaglandin and oxytocin. Besides, the low station of the fetal head suggests that women are more likely to have a favorable bishop score, which may result in a successful TOLAC [40].

The odds of having a successful TOLAC was higher in a women had history of vaginal birth before the first CS and this study finding was similar with other studies [24, 30, 34, 41].

The possible explanation could be the fact that the maternal pelvis was tested for vaginal delivery and decreased fear of labor pain and childbirth. The other justification might be the fact that the Women with a history of vaginal birth may have a better awareness of the benefits of vaginal delivery than CS [41].

The current study also found that previous successful TOLAC was substantially associated with the current success of TOLAC. This was consistent with various study findings [22, 24, 27, 41]. This could be explained as a woman who have had previous success with TOLAC may be mentally prepared and aware of the benefits of vaginal delivery. Having prior TOLAC suggests that the cause of primary CS is non-recurring, which may help the health care professional refrain from making an early decision on the mode of delivery.

It has been also stated that the history of VBAC facilitates the progress of labor, decreases the risk of subsequent uterine rupture, and reduces the fear of women and health professionals about TOLAC [39].

Note that, cautious selection of candidates for TOLAC, close and ongoing intrapartum maternal and fetal monitoring plays a significant role to reduce the risk of uterine rupture and other related complications such as hysterectomy, blood transfusion and anesthesia related complications [42]. A suitable prediction model could be clinically useful in identifying women who are more likely to have a successful TOLAC [43], Finally, it’s strongly suggested that providing antenatal and intrapartum information and counselling to the women is fundamental to make informed decision on trial of labor after cesarean section. The clinicians must provide attentive monitoring and supportive care during the throughout stages of labor to achieve a successful trial of labor after cesarean section, and alternative options should be ready for emergency situations.

### Strengths and limitation of the study

The study was conducted in five randomly selected tertiary hospitals, which increases the variability of TOLAC cases and the generalizability of the findings. However, our study also had some limitations. Due to its cross-sectional nature, it's difficult to determine causal relationships between the dependent and predictor variables. It’s good to conduct further randomized control trials and prospective studies to determine the exact association between the success of TOLAC and different independent variables.

## Conclusion

The success rate of TOLAC in this study area was low as compared to the ACOG guideline and many studies conducted in different countries. A woman with advanced age (≥ 35 years), low fetal station on admission (≤ 0), having vaginal birth prior to the first CS, and history of prior successful VBAC were found to be a good candidate for successful TOLAC. Therefore, the clinicians ought to offer counsel during antenatal and intrapartum period, encourage the women to make informed decision on the mode of delivery, and the practitioners need to follow fetal and maternal conditions strictly to minimize adverse birth outcomes.

## Data Availability

The data sets used for this research are available from the corresponding author upon reasonable request.

## Abbreviations

ACOG: American College of Obstetrician and Gynecology
AOR: Adjusted Odds Ratio
CI: Confidence Interval
COR: Crude Odds Ratio
CS: Caesarean Section
TOLAC: Trial of Labor After Caesarean Section
VBAC: Vaginal Birth After Caesarean Section
